# Accessory Spleen Mimicking an Intrahepatic Neoplasm: A Rare Case Report

**DOI:** 10.7759/cureus.39185

**Published:** 2023-05-18

**Authors:** Peethambaran M S, Catherine Matthew, Rajkamal R Rajendran

**Affiliations:** 1 Gastrointestinal Surgery, Avitis Institute of Medical Sciences, Palakkad, IND; 2 Pathology, Avitis Institute of Medical Sciences, Palakkad, IND

**Keywords:** ectopic spleen, liver lesion, splenosis, intra hepatic spleen, accessory spleen

## Abstract

Accessory spleen and splenosis are two types of ectopic spleen. An accessory spleen can be found in various sites in the abdomen, but an intrahepatic accessory spleen is very rare though many case reports of intrahepatic splenosis are available.

This case report presents the incidental diagnosis of accessory spleen in the liver of a 57-year-old male while undergoing laparoscopic diaphragmatic repair. The patient had a history of splenectomy 27 years ago for hereditary spherocytosis, but his routine haemogram did not show any features of the ectopic splenic function. Intraoperatively, a mass was suspected in the liver and was resected. Histopathology revealed an accessory spleen with well-preserved red and white pulp architecture. Though a history of splenectomy suggested a diagnosis of splenosis, a well-encapsulated and preserved splenic architecture confirmed the diagnosis of accessory spleen.

Accessory spleen or splenosis can be diagnosed radiologically using Tc-99m-labeled heat-denatured red blood cells (HRBC) and Tc-99m sulfur colloid scans, but the gold standard is histopathological examination. Ectopic spleen is mostly asymptomatic but usually results in unnecessary surgeries as it is difficult to differentiate from benign or malignant tumors. Thus, a high degree of suspicion and awareness is necessary for early and prompt diagnosis.

## Introduction

Ectopic splenic tissue occurs in two different forms - accessory spleen and splenosis. Accessory spleen occurs due to developmental anomalies. Splenosis occurs due to trauma or surgery where some portion of the splenic tissue gets incorporated in the peritoneal cavity and receives local blood supply and becomes functional splenic tissue [1] An accessory spleen is located most commonly in the splenic hilum (75%) followed by the tail of the pancreas (25%). It is also found in the gastrosplenic ligament, splenorenal ligament, wall of the stomach, bowel, greater omentum, mesentery, and even in the pelvis and scrotum [2]. Both are diagnosed more commonly in patients who had undergone therapeutic splenectomies as the ectopic splenic tissue enlarges and starts to show splenic functions.

The present case report is a rare case of accessory spleen located in the liver diagnosed incidentally during a diaphragmatic hernia repair in a 57-year-old male with a history of splenectomy for Hereditary Spherocytosis 27 years ago.

## Case presentation

A 57-year-old male presented to the Emergency Department with a history of abdominal distension and feeling of incomplete evacuation of bowel for ten days. He is a diabetic and hypertensive on regular medications. In 1995, he underwent a laparoscopic cholecystectomy along with an open splenectomy for Hereditary Spherocytosis. He also underwent ureteroscopic lithotripsy in 2018 and developed COVID-19 pneumonia in 2021.

A general examination of the patient revealed no pallor, a heart rate of 98 beats /minute, blood pressure of 150/111mmHg, oxygen saturation of 99% in room air, blood sugar of 194mg/dl, and body temperature of 98.6F.

Systemic examination showed a soft abdomen with sluggish bowel sounds and a surgical scar of the previous left subcostal incision. Hematological investigations showed blood urea levels at 26 mg/dl with borderline creatinine (1.3mg/dl), sodium at 137 mEq/L, potassium at 4.1 mEq/L, C-reactive protein at 12mg/l, and Hb at 13.4 g/dl. The total leucocyte count was elevated with 13600 cells /cu mm, with a normal differential count and a platelet count of 3.66 x10^5^.

As the patient showed features of partial intestinal obstruction, a plain abdominal CT was done. Intravenous contrast was not given as his renal parameters were borderline. CT showed focal dilated mid-ileal loops with air-fluid level, a left hemi diaphragmatic hernia with herniation of gastric fundus and omental fat, and a small right kidney with a focal area of cortical scarring.

The patient was managed conservatively for intestinal obstruction with nil per mouth, intravenous fluids, and other supportive medications. The patient improved with medical management and was discharged. He was reviewed in the outpatient department on day 19 and day 71 after the initial presentation with abdomen fullness and excessive sound from the abdomen.

As the patient had a diaphragmatic hernia and mild symptoms of bowel adhesions persisting, elective laparoscopic adhesiolysis with a repair of the hernia was planned. Preoperative investigations were essentially normal except for elevated potassium which was 5.7 meq/l, while renal function tests were within normal limits before surgery and the treadmill test was negative at 10 METS.

**Figure 1:**
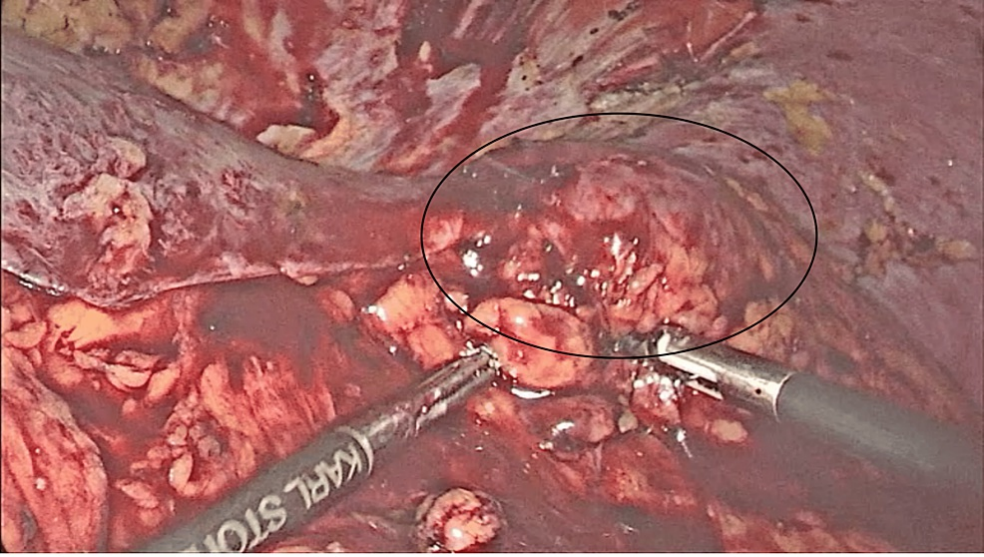
Suspected mass in left lateral segment of the liver

Surgery was performed under general anesthesia. Intraoperatively, while mobilizing the liver, a mass lesion was suspected in the lateral segment of the left lobe of the liver (segment 2/3) (Figure 1).

**Figure 2:**
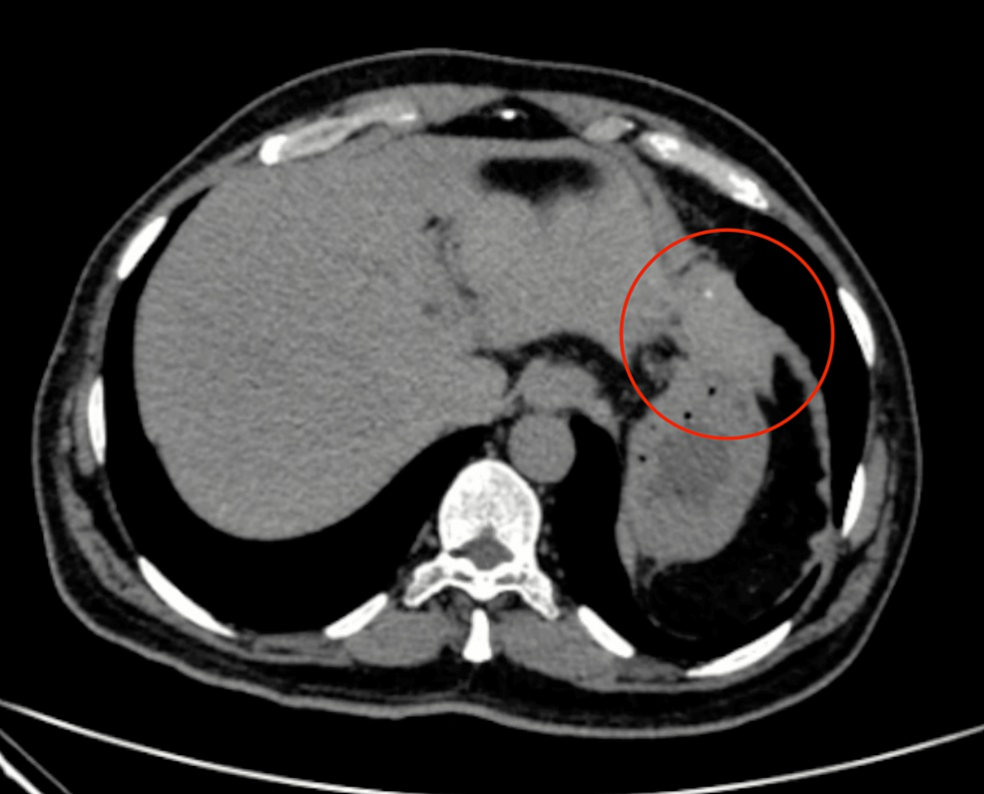
The suspected area on CT

**Figure 3:**
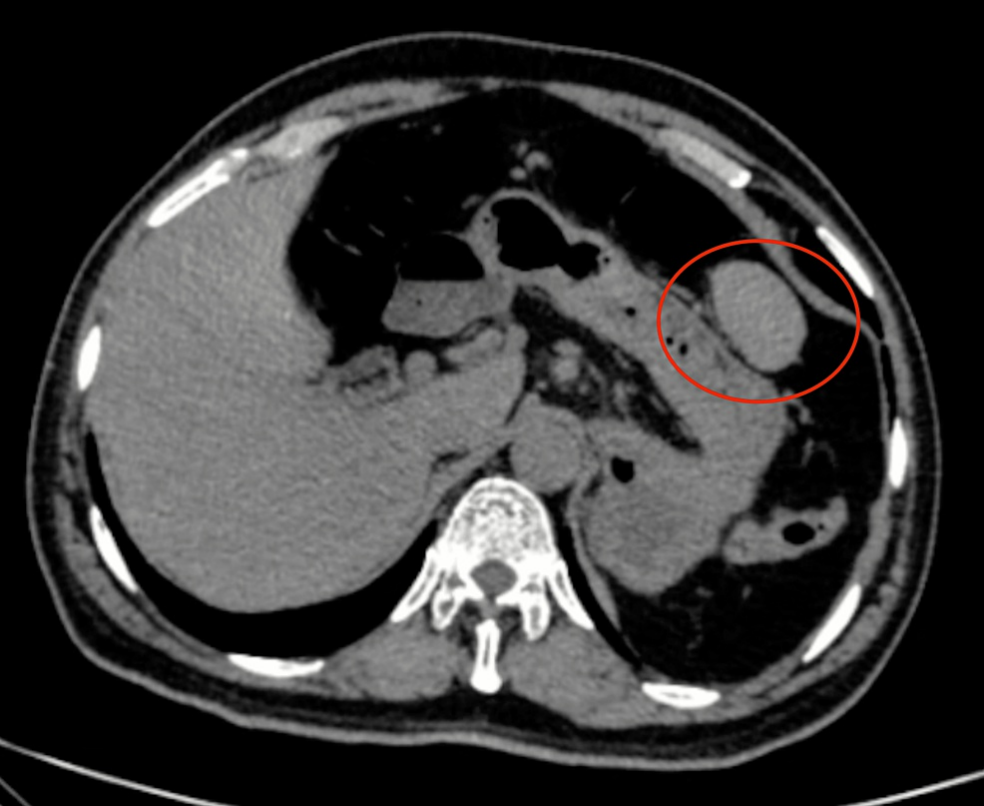
Lower cut of the same CT showing the lesion

A review of the previous CT was done and discussed with the radiologist, and a conclusion of a suspicious mass was made (though not contributory as it was a non-contrast film) (Figure 2, 3).

**Figure 4:**
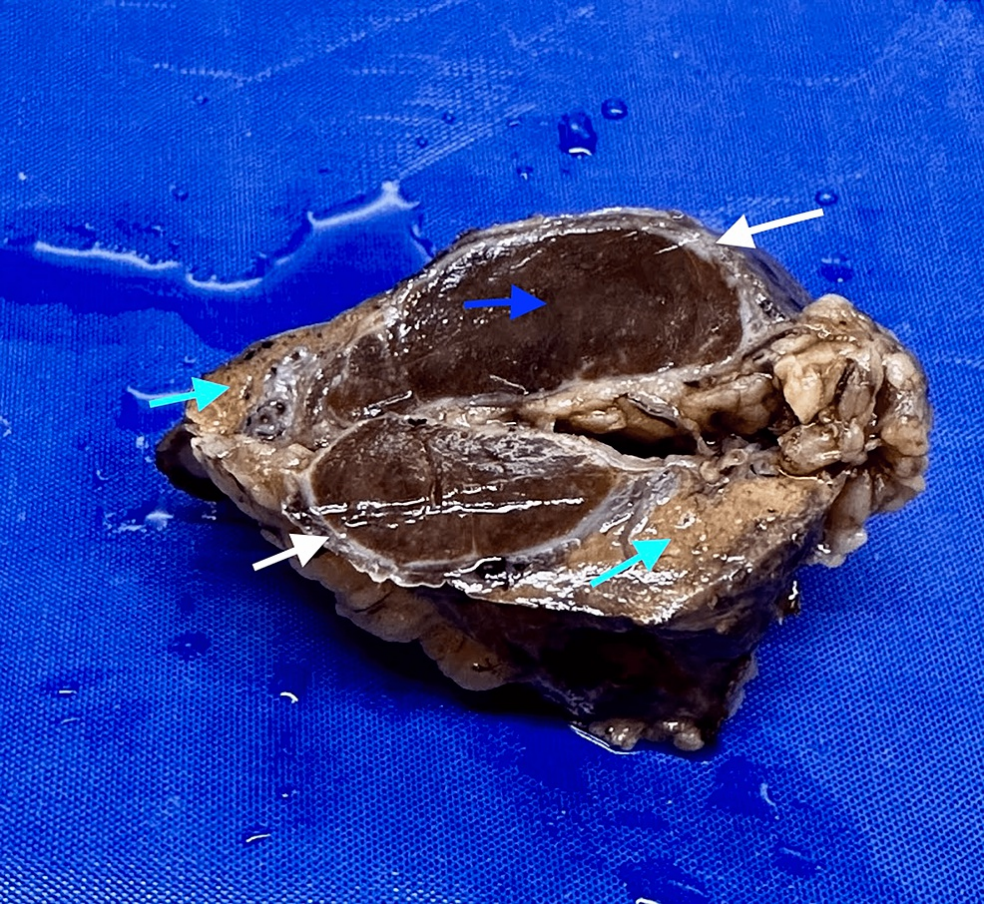
Cut section of the specimen showed an intrahepatic firm oval brown-colored lesion measuring 3x1.2 cm Green arrows- Liver tissue; white arrows- splenic capsule; blue arrow- splenic tissue

Due to the non-availability of a laparoscopic intraoperative ultrasound and after discussing with relatives and obtaining consent, it was decided to convert to laparotomy and proceed. Laparotomy was done through the previous left subcostal incision. Non-anatomic resection of the liver-containing mass was done, along with diaphragmatic hernia repair and adhesiolysis. Reinforcement of the laparotomy was done with a mesh as the scar was very weak. A cut section of the specimen showed an intrahepatic firm oval brown-colored lesion measuring 3x1.2 cm (Figure 4).

**Figure 5:**
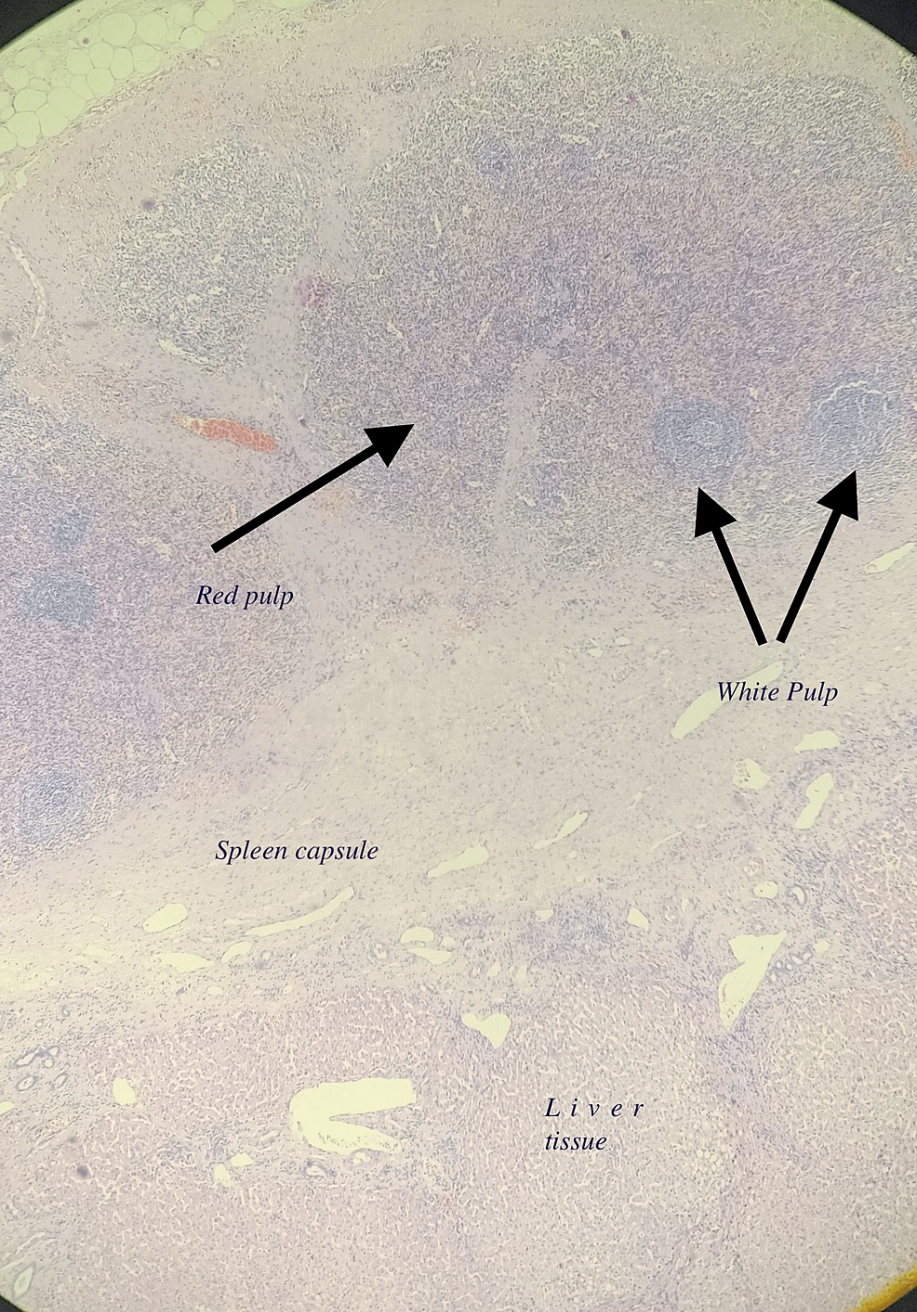
Low magnification view showing Liver tissue in the lower part and splenic tissue with preserved architecture and well-defined capsule

The patient was extubated at the end of the surgery. Postoperatively, the patient had derangement of renal function tests which normalized with conservative management. The patient was discharged on postoperative day seven. Histological analysis revealed a well-circumscribed and encapsulated lesion with preserved white and red pulp architecture, which was suggestive of accessory spleen. Figure 5 shows a preserved splenic architecture and capsule, and the liver tissue.

**Figure 6:**
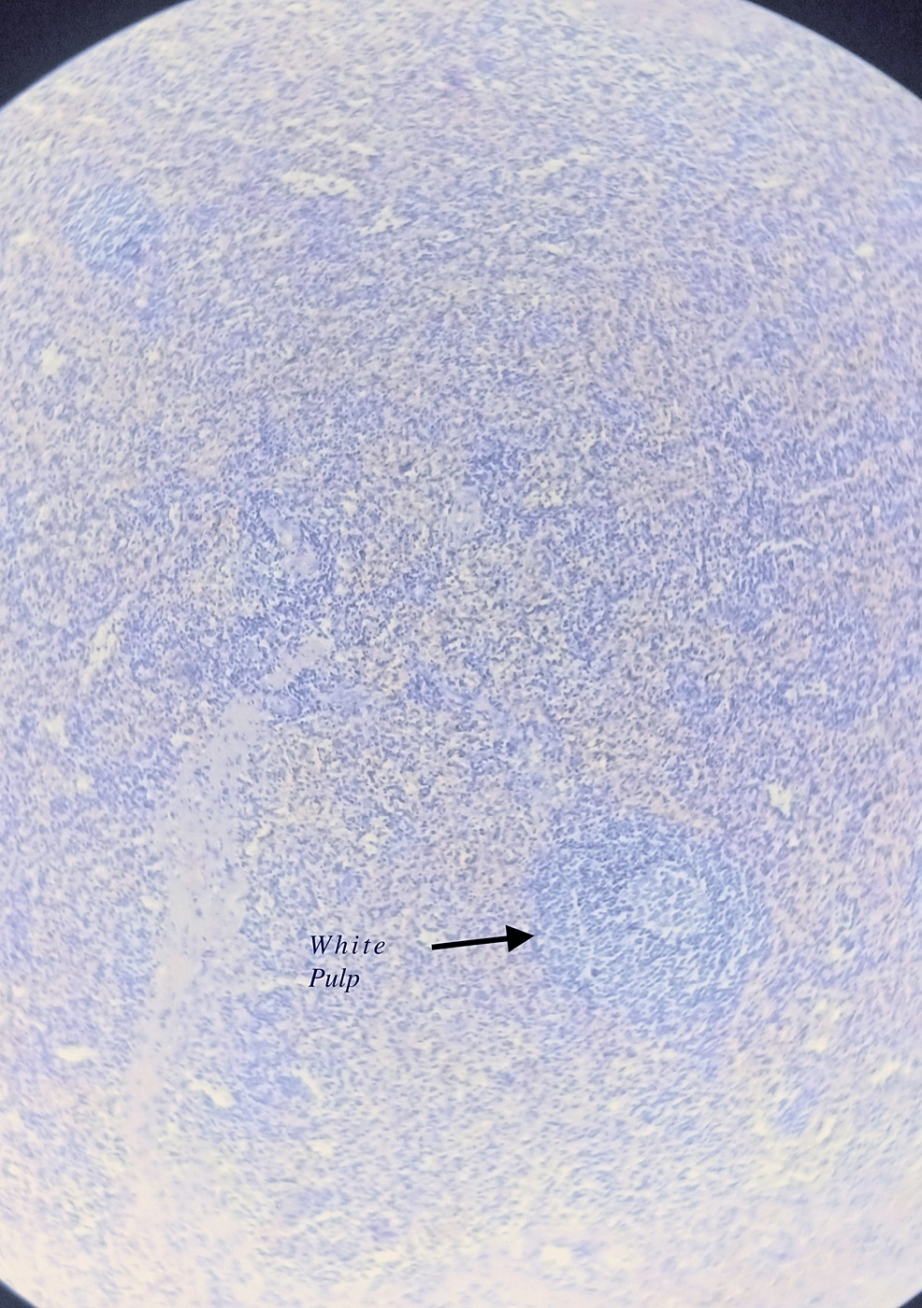
High magnification view showing red and white pulp

Figure 6 shows the view of the splenic part of the histology at a higher magnification. The red and white pulp can be seen

## Discussion

An accessory spleen arises due to the failure of fusion of multiple splenic analges in the dorsal mesogastrium during the fifth week of embryonic development [3][4]. A meta-analysis and systematic review of 22,487 patients by Vikse et al. showed the prevalence of accessory spleen to be 14.5% which includes imaging, intraoperative, and cadaveric studies, and is more in patients with immune thrombocytopenic purpura [5]. An accessory spleen can be single or multiple. It is a round or oval structure with an average diameter ranging from 1-3 cm [6][7].

An accessory spleen is mostly asymptomatic but can present with infarction, hemorrhage, torsion, and rupture [8]. Usually, it is diagnosed as an incidental finding during radiology or surgery. Most cases of accessory spleen are misdiagnosed as tumors or lymph nodes which leads to unnecessary surgical removal [9]. It can also lead to the reappearance of hematological symptoms where splenectomy was done for therapeutic purposes. This patient did not have any hepatic symptoms or any features of a functional spleen even though he underwent splenectomy for Hereditary Spherocytosis. 

An intrahepatic spleen does not need any resection if it is asymptomatic. In this case, we proceeded with resection as the possibility of malignancy could not be ruled out. Our patient was admitted for a diaphragmatic hernia repair and adhesiolysis. Despite the open splenectomy for Hereditary Spherocytosis, his laboratory parameters did not show any features of any functional accessory spleen. Though the presence of an accessory spleen is relatively common, its intrahepatic location is very rare. Most of the cases of intrahepatic spleen described in the literature are splenosis rather than an accessory spleen.

Izzo et al. [10] have described a case of an intrahepatic accessory spleen in a 60-year-old chronic hepatitis patient confirmed by ultrasound-guided biopsy. Contrast-enhanced CT was not performed on our patient as he had borderline renal parameters. However, the characteristic imaging of an accessory spleen is not specific and almost indistinguishable from other hepatic neoplasms, especially hepatocellular carcinoma (HCC). The gold standard for diagnosis of an accessory spleen or splenosis is histopathological examination. However, this requires unnecessary excision or biopsy of the lesions. The non-invasive method of choice is Tc-99m-labeled heat-denatured red blood cells (HRBC), and Tc-99m sulfur colloid scans. Soliman et al. [11] have reported a case of intrahepatic splenosis with a false negative Tc-sulphur colloid scan due to lymphomatous transformation of the splenic tissue.

Histologically, the spleen can display various developmental anomalies including complete agenesis to polysplenia. Ectopic splenic tissue can be either congenital (accessory spleen or splenunculi ) or acquired (splenosis) [12]. An accessory spleen has normal splenic tissue histology and is supplied by the branches of the splenic artery. However, splenosis is an acquired condition, has distorted microscopic architecture, and is supplied by surrounding vessels [4] 

In our patient, sections studied show a well-circumscribed lesion composed of splenic tissue with preserved white pulp and red pulp. This tissue was separated from the adjacent liver tissue by a well-defined capsule. The presence of a solitary lesion with normal splenic architecture and a capsule separating the splenic tissue from hepatic parenchyma made us consider this as an ectopic hepatic spleen/accessory spleen [13]. The absence of Howell- Jolly bodies in patients post-splenectomy indicates the presence of an ectopic spleen [14].

## Conclusions

Intrahepatic spleen, though rare, should be considered in the differential diagnosis of hepatic lesions, especially in patients who have undergone a splenectomy. Awareness and a high degree of suspicion, along with adequate imaging, can prevent unnecessary hepatic resections. In post-splenectomy patients, the presence of Howell-Jolly bodies may be indirect evidence of the absence of ectopic spleen.
